# Métodos empleados para cuantificar la carga de trabajo en Enfermería en las unidades de cuidados intensivos: Una revisión de la literatura

**DOI:** 10.15649/cuidarte.2301

**Published:** 2023-03-31

**Authors:** Diana Isabel Cáceres-Rivera, Jessica Paola Ruiz-Sandoval, Luisa Yaneth Cristancho-Zambrano, Maria Andreina Pulido-Montes, Luis Alberto López-Romero

**Affiliations:** 1 Enfermera, Magister en Enfermería, Doctora en Biomedicina. Universidad Cooperativa de Colombia. Facultad de Enfermería. Bucaramanga. Colombia. E-mail: dianai.caceres@ucc.edu.co Universidad Cooperativa de Colombia Universidad Cooperativa de Colombia Colombia dianai.caceres@ucc.edu.co; 2 Enfermera. Universidad Cooperativa de Colombia. Facultad de Enfermería. Bucaramanga. Colombia. E-mail: jrsandoval9618@gmail.com Universidad Cooperativa de Colombia Universidad Cooperativa de Colombia Colombia jrsandoval9618@gmail.com; 3 Enfermera. Fundación Cardiovascular de Colombia- Hospital Internacional de Colombia. Unidad de Cuidado Intensivo. Bucaramanga. Colombia. E-mail: luisa.cristancho0217@gmail.com Fundación Cardiovascular de Colombia Colombia luisa.cristancho0217@gmail.com; 4 Enfermera, Magister en Enfermería. Universidad Cooperativa de Colombia. Facultad de Enfermería. Bucaramanga. Colombia. E-mail: mariaa.pulido@ucc.edu.co Universidad Cooperativa de Colombia Universidad Cooperativa de Colombia Colombia mariaa.pulido@ucc.edu.co; 5 Enfermero Magister en Epidemiologia. Egresado Escuela de Enfermería Universidad Industrial de Santander. Bucaramanga. Colombia. E-mail: alberlop60@gmail.com Universidad Industrial de Santander Universidad Industrial de Santander Colombia alberlop60@gmail.com

**Keywords:** Enfermería, Atención de Enfermería, Carga de trabajo, Medición de Riesgo, Unidades de Cuidados Intensivos., Nursing**,** Nursing Care, Workload, Risk Assessment, Intensive Care Units, Enfermagem, Cuidados de Enfermagem, Carga de Trabalho, Medigáo de Risco, Unidades de Terapia Intensiva.

## Abstract

**Introducción:**

La carga de trabajo de Enfermería en unidades de cui dado intensivo está relacionada con la eficiencia y calidad de la aten ción, sin embargo, no existen métodos para cuantificar las enfermeras necesarias por turno en UCI.

**Objetivo:**

Identificar las herramientas más utilizadas para medir la carga de trabajo de Enfermería en UCIs.

**Materiales y métodos:**

Se realizó una revisión de literatura tipo integradora, utilizando artículos originales en inglés, español o portugués, publica dos entre 1991 hasta 2017 en las bases de datos: Science@direct, BVS, Socupus y Embase, empleando la estrategia de búsqueda: *Nursing and workload and intensive critical or ICU unit and measure*, se excluyeron ar tículos duplicados y/o desarrollados en UCIs de cuidado intermedio, la calidad de los artículos fue valorada usando la lista de chequeo Strobe.

**Resultados:**

Se incluyeron 36 artículos con un total de 19.036 pacien tes; el 50% (n=18) empleo el NAS, 27.7%(n=10) utilizó una combinación de métodos como el NAS, NEMS, TISS-28 o el VACTE, el 13.8%(n=5) em pleo el TISS-28, el 5.6%(n=2) empleo registro de cámaras de video y un 2.7%(n=1) empleo el NEMS para cuantificar el tiempo empleado por en fermería en el cuidado.

**Discusión:**

actualmente no existe un consenso sobre métodos de medición de carga de trabajo en enfermería, en este sentido, es necesario realizar más estudios de validación y compara ción que permitan mejorar la gestión del cuidado de enfermería en UCI.

**Conclusión:**

La herramienta más utilizada para cuantificar la carga de trabajo en enfermería es el Nursing Activities Score (NAS), otras herra mientas identificadas fueron: NEMS, TISS-28 y VACTE.

## Abstract (en)

**Introduction:**

Nursing workload in intensive care units (ICUs) is related to efficiency and quality of care; however, there are no methods to quantify the nurses needed per shift in ICUs.

**Objective:**

To identify the most used tools to measure ICU nursing workload.

**Methods:**

An integrative literature review was performed using original articles in English, Spanish or Portuguese, pub lished between 1991 and 2017 in the databases ScienceDirect, BVS, Scopus, and Embase. The search strategy was “*nursing and workload and intensive critical or ICU and measure.*” Duplicate articles or articles about intermediate care units were excluded. The quality of the articles was assessed using the Strobe checklist.

**Results:**

Thirty-six articles with a total of 19,036 patients were included; 50% (n=18) used NAS, 27.7% (n=10) used a combination of methods such as NAS, NEMS, TISS-28 or VACTE; 13.8% (n=5) used TISS-28, 5.6% (n=2) used video camera record ing, and 2.7% (n=1) used NEMS to quantify the time spent by nurses in care.

**Discussion:**

There is currently no consensus on workload measurement methods in nursing. In this sense, more validation and comparison studies are needed to improve nursing care management in the ICUs.

**Conclusion:**

The most used tool to quantify workload in nursing is the Nursing Activities Score (NAS). Other tools identified were NEMS, TISS-28, and VACTE.

## Resumen

**Introdujo:**

A carga de trabalho de enfermagem em unidades de terapia intensiva está relacio nada a eficiencia e qualidade da assistencia, entretanto, nao existem métodos para quantificar o número de enfermeiros necessários por turno na UTI.

**Objetivo:**

Identificar os instrumentos mais utilizados para mensurar a carga de trabalho de Enfermagem nas UTIs.

**Metodologia:**

Foi realizada uma revisao integrativa da literatura, utilizando artigos originais em ingles, espanhol ou portugues, publicados entre 1991 e 2017 nas bases de dados: Science@direct, BVS, Socu- pus e Embase, utilizando a estratégia de busca*: Nursing and workload and intensivo critical* ou unidade e medida de UTI, artigos duplicados e/ou desenvolvidos nas UTIs de cuidados inter- mediários foram excluídos, a qualidade dos artigos foi avaliada por meio do Strobe checklist.

**Resultados:**

foram incluídos 36 artigos com um total de 19.036 pacientes; 50% (n=18) utiliza- ram o NAS, 27,7%(n=10) utilizaram uma combinado de métodos como NAS, NEMS, TISS-28 ou VACTE, 13,8%(n=5) utilizaram o TISS-28, 5,6% (n=2) utilizaram registros de cameras de vídeo e 2,7%(n=1) utilizaram o NEMS para quantificar o tempo gasto pela enfermagem no cuidado.

**Discussao:**

atualmente nao há consenso sobre métodos de mensurado da carga de trabalho em enfermagem, nesse sentido, faz-se necessária a realizado de mais estudos de validagáo e comparado para melhorar o gerenciamento do cuidado de enfermagem em UTI.

**Conclusao:**

O instrumento mais utilizado para quantificar a carga de trabalho de enfermagem é o *Nursing Activities Score* (NAS), outros instrumentos identificados foram: NEMS, TISS-28 e VACTE.

## Introducción

Las tareas asignadas de acuerdo al sitio de desempeño de las enfermeras son variables y ge neralmente están distribuidas acorde a políticas institucionales. En las unidades de cuidado intensivo, la enfermera debe distribuir sus labores en actividades administrativas, asistenciales y de gestión. Con relación a lo anterior, no existe un consenso para determinar la cantidad de enfermeras necesarias en una unidad de cuidado intensivo. Sin embargo, se acepta, en general, que a medida que aumentan las tareas de un individuo, mayor es el riesgo que se corre de in cumplir algunas, o de disminuir la eficiencia y calidad final. Así lo aseguran algunos reportes que sostienen que por cada paciente adicional asignado a una enfermera se aumenta la mortalidad entre otras situaciones^1^.

En concordancia con lo anterior y al no haber una recomendación del número de Enfermeras por cada paciente de en cuidado intensivo, el tiempo de cuidado directo de enfermería se ha disminuido. Adicionalmente, esta falta de tiempo al generar una carga adicional y limitar el con tacto directo con el paciente, debilita las barreras de seguridad con lo que se pueden presentar no solo inconformidades en la calidad del cuidado si no una disminución de los indicadores de calidad de una institución^2^. Algunos estudios han demostrado por ejemplo que no contar con el personal de enfermería suficiente pone en peligro la calidad de la atención del paciente, lo que incrementa a su vez la incidencia de infecciones, lesiones por presión, complicaciones postope ratorias e incremento de la estancia hospitalaria y muerte^3^.

Las unidades de cuidado intensivo son servicios con los que cuentan las instituciones pres tadoras de salud de tercer nivel con el fin de proveer la atención de más alta calidad y deben contar con la infraestructura y personal necesarios para satisfacer la demanda de cuidados que se incrementa comparativamente en relación con otros servicios hospitalarios. En ese contexto, y dadas las condiciones clínicas críticas de los pacientes que se requiere de un mayor tiempo, conocimiento y entrenamiento para proveer atención de calidad, siendo preponderante una relación enfermera-paciente racional en términos de calidad y satisfacción de la demanda de cuidados^4^^,^^5^.

Con respecto a la carga de trabajo en enfermería en unidades de cuidado intensivo, algunos in vestigadores han tratado de contextualizar este concepto, ya sea basándose en la agudeza del paciente, complejidad de la atención, gravedad de la enfermedad, dependencia o tiempo de cuidado directo. Esto ha generado la aparición de los términos trabajo y carga de trabajo de en fermería, usualmente empleados para describir el mismo concepto, aunque tienen significados diferentes; con respecto a la carga de trabajo en enfermería se han descrito aquellas estrategias que evalúan la cantidad de tiempo que toma completar tareas que deben ser realizadas duran te un tiempo dado^6^. Sin embargo, una definición más completa la describe como el tiempo ne cesario para llevar a cabo la atención directa e indirecta del paciente, así como otras actividades de organización y gestión, esta definición considera que el concepto está conformado por más factores que las características del paciente^7^.

En este sentido y durante más de 30 años en un intento de demostrar la relación costo-benefi cio de la unidad de cuidados intensivos (UCI) se ha desarrollado una variedad de herramientas para medir no solo la gravedad de la enfermedad del paciente, sino también para calcular el costo real de la carga de trabajo en enfermería^8^. Algunos de estas herramientas de medición se han basado en el paciente o en el cuidador. Aquellas centradas en el paciente tienen en cuenta características como el número de pacientes, la relación enfermera - paciente, la agudeza de la enfermedad o el tiempo de atención. Por el contrario, la medición de carga de trabajo en elcuidador considera características de la enfermera, así como sus interacciones con su entorno de trabajo^9^.

Algunos autores como Gonalves y col, describieron algunas de las herramientas que se han creado desde 1981 para tal fin. Es así como menciona entre otros el PRN (Project of research of Nursing), el OMEGA (Omega Scoring System), el TOSS (Time Oriented Score System), el SOPRA (Systema of Patient Related Activity), el TISS-28 (Therapeutic Intervention Soring System), el NEMS (Nine equivalents of Nursing Manpower Score) y el NAS (Nursing Activities Score^10)^. Debi do a la variedad de herramientas que existen y al desarrollo continuo que ha tenido Enfermería en este tema creando nuevas posibles herramientas para la medición de la carga de trabajo en Enfermería en las Unidades de Cuidado Intensivo, esta revisión sistemática tuvo como objetivo identificar las herramientas más utilizadas para medir la carga de trabajo en enfermeras en las Unidades de Cuidados Intensivos.

## Materiales y Métodos

Esta investigación fue realizada bajo criterios de una metodología de revisión integrativa de la literatura, con el objetivo de identificar las herramientas más utilizadas para medir la carga de trabajo de Enfermería en Unidades de Cuidado Intensivo. En ese sentido, el presente estudio direcciono un alcance de búsqueda, selección y revisión de artículos originales y con disponibi lidad de textos completos en inglés, español o portugués, publicados entre el año 1991 hasta 2017 con el fin de evaluar de forma crítica y a su vez sintetizar el estado actual del conocimiento sobre el tema investigado^11^.

Se llevó a cabo una revisión de la literatura tipo integradora en las siguientes bases de datos: Embase, Biblioteca Virtual de Salud de la OMS (BVS), Sciencedirect y Scopus. Adicionalmente se realizó una indagación manual con los resultados de la búsqueda original en las bases de datos antes mencionadas. Esta se realizó empleando la siguiente sintaxis de búsqueda: *Nursing and workload and intensive critical or ICU unit and measure.* Lo anterior con la finalidad de poder responder a la siguiente pregunta de investigación: ¿Cuáles son los métodos empleados para medir el tiempo de las actividades asistenciales, administrativas y de gestión de una enfermera en las Unidades de Cuidados Intensivos?

Los resultados de la búsqueda fueron filtrados para descartar los artículos duplicados. Así mis mo, se tuvo en cuenta que la población fueran sujetos adultos internados en unidades de cui dado intensivos y que evaluaran el tiempo del personal profesional de Enfermería; esto con el objetivo de conocer los métodos y tiempo empleados por el personal de enfermería en cada una de las actividades (asistenciales, administrativas y de gestión) en la unidad de cuidados in tensivos. Se excluyeron los artículos cuyas UCIs incluidas correspondían a Unidades de cuidado intermedio. De igual forma se excluyeron revisiones narrativas o de tema, artículos de valida ción, de opinión o reflexión, editoriales y presentaciones en congresos, conferencias o blogs.

Inicialmente, todos los resúmenes de artículos potencialmente relevantes fueron revisados. Posteriormente se desarrollaron las 5 etapas propuestas por Whittemore y Knafl: 1. Identifica ción del problema, 2. búsqueda de literatura, 3. evaluación de los datos, 4. análisis de los datos y 5. presentación de resultados^12^.

**Figura 1:**
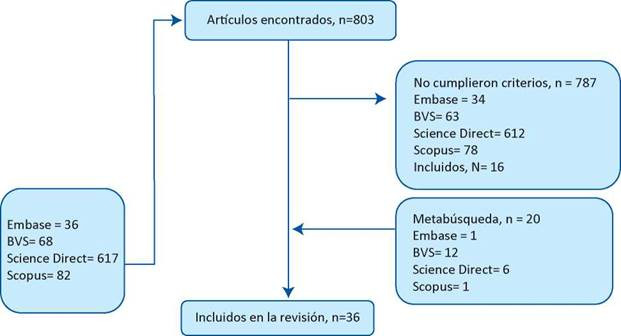
Flujograma de artículos encontrados según la literatura revisada. Bucaramanga-Colombia 2018

En ese sentido, sí cumplía con los criterios de inclusión mencionados, el artículo era analizado por los investigadores utilizando la lista de chequeo Strobe para artículos observacionales a fin de evaluar su calidad. Posteriormente, se realizaba una extracción y clasificación de los datos más relevantes de forma independiente por dos miembros del equipo, luego investigadores principales realizaban el doble chequeo de la información obtenida por los ítems de Strobe. En la figura 1, se presenta el flujograma de la búsqueda y los estudios incluidos. Finalmente, se elaboró una tabla resumen a través de medidas de tendencia central y frecuencias absolutas y relativas con los principales métodos, así como el tiempo empleado en cada una de las activida des, con el objetivo de identificar de manera eficaz las posibles conclusiones, las cuales fueron sintetizadas una vez finalizada la revisión de las fuentes bibliográficas.

## Resultados y Discusión

Se encontraron un total de 803 artículos con la estrategia de búsqueda definida previamen te, de los cuales 787 fueron excluidos al no cumplir los criterios de inclusión o debido a que estaban duplicados en las diferentes bases de datos. Es así como 16 artículos cumplieron con los criterios de búsqueda y 20 fueron incluidos por proceso de meta búsqueda a partir de los artículos originales.

Finalmente 36 artículos fueron incluidos en la presente revisión, como se aprecia en la Figura 1. De acuerdo con la lista de comprobación de estudios observacionales, los 36 artículos incluye ron de manera completa y clara los apartados necesarios para considerar su calidad, incluyendo una adecuada descripción del tipo de metodología y análisis de los resultados. Se identificaron diferentes estudios de tipo transversal, longitudinal, retrospectivo, predominando los de tipo prospectivo. Las demás características corresponden a las tenidas en cuenta en los criterios de inclusión como haber sido realizados en Unidades de Cuidado intensivo con profesionales de enfermería, haber utilizado un instrumento para medición de carga laboral, entre otras. Es im portante resaltar que la muestra incluida en los artículos revisados, tuvo una gran variabilidad que dependía de la institución en donde fue realizada y por ende del número de enfermeros que trabajaban en cada una de las unidades evaluadas.

De los 36 artículos incluidos en la presente revisión, el 50% (n=18) empleo el NAS para cuantifi- car el tiempo de las actividades de enfermería, el 27.7%(n=10) empleo una combinación de dos o más métodos entre los cuales estaban el NAS, NEMS, TISS-28 o el VACTE, 13.8%(n=5) empleo le TISS-28 o una versión modificada. Un 5.6%(n=2) empleo registro de cámaras de video y un 2.7%(n=1) empleo el NEMS para cuantificar el tiempo dedica por enfermería a las diferentes labores de cuidado, Tabla 1.

El presente artículo permitió describir las diversas herramientas disponibles para evaluar la car ga de trabajo de enfermería en las Unidades de cuidado intensivo. Una de estas corresponde, a la evaluación a través de la observación directa. Igualmente, algunas unidades de acuerdo con sus necesidades han creado instrumentos de medición que sin embargo no cuentan con un proceso de validación completo.

Los resultados anteriormente descritos guardan relación con el proceso que se ha desarrollado en Enfermería con el fin de encontrar la herramienta ideal que permita identificar la carga labo ral de las Enfermeras en UCI. En este sentido, el NAS (Nursing Activities Score) es aquel que más ha sido utilizado, ha demostrado ser el que cuenta con mayor número de estudios. (Tabla 1). Es necesario describir que este instrumento es resultado de la evolución de varios instrumentos hallados en la literatura como el TOSS y el TISS 28 (Therapeuthic Intervention Scoring System) los cuales se enfocaban más en describir la gravedad de los pacientes que en la carga de tra- bajo^13^. Sin embargo, aún se utiliza este último para calcular la carga de trabajo de enfermería en unidades de cuidado intensivo. De hecho, otro hallazgo interesante tiene que ver con el in cremento gradual de estudios donde se realiza la comparación que se ha hecho de otro instru mento como lo es el NEMS (Nine equivalents of nursing manpower use score) con el TISS-28 y el NAS. Estos tres fueron desarrollados por Miranda et al, con 6 años de diferencia, siendo el más reciente el NAS. Sin embargo, dentro de los artículos revisados, los más recientes corresponden a resultados de investigaciones donde utilizaron el TISS-28.

Estos hallazgos, son acordes a lo expuesto por Fajardo Quintana y Cols quien refiere que: “La complejidad de los cuidados de enfermería y el avance tecnológico, relacionados con el pacien te crítico, han puesto de manifiesto la necesidad de revisar y actualizar los sistemas de cuantifi- cación de cargas de trabajo”^14^.

En este sentido, la presente revisión demuestra que, si bien hay una amplia divulgación de los diversos instrumentos de medición de carga laboral en enfermería en unidades de cuidado intensivo, no existe un consenso sobre cuál y porque aplicar. Incluso, se encontraron artículos con instrumentos o tipos de medición nuevos y con limitados estudios de validación. Es por esta razón que se deben realizar un mayor número de estudios de validación y comparación en diferentes contextos culturales con el fin de tener un acercamiento a herramientas confiables que permitan gestionar el cuidado de enfermería en UCI de la mejor manera.

**Tabla 1:** Artículos relacionados con cuantificación de carga de trabajo de Enfermería utili zando NAS, TISS-28, NEMS y otras herramientas

Nursing Activities Score					
Autor/Año	Población (n)	Diseño	Método	Resultado	Otras variables de interés
Armstrong y col, 2015^15^.	87 pacientes	Corte transversal	NAS	El promedio del NAS fue comparado durante el día, tarde y noche para la UCI intermedia versus la UCI completa: 43.9±13.2 Vs 41.8±11.0; 44.3 ±12.8 Vs 43.1 ± 10.0 y 37.0±12.0 Vs 32.7±9.3, respectivamente	N A
Bernat y col, 2005^16^.	350 pacientes	Descriptivo longitudin al	NAS	NAS promedio de 41.27±10.80. El primer día fue 40.8 ±14.1 Vs alta: 39.3 ± 12.7	4.3±5.4 días de estancia
Cardoso de Sousa y col, 2009^17^.	600 pacientes	Prospectivo longitudin al	NAS	El NAS al ingreso versus el egreso para cada población fue: Adultos, 59.98 ±22.40% Vs 50.37± 14.56, ancianos: 64.41±20.66 Vs 55.85±16.77 y muy ancianos: 62.45±20.80 Vs 53.39±16.17	8.90±10.90 días de estancia 20.0% mortalidad
Carmona - Monge y col, 2012^18^.	103 pacientes	Estudio prospectivo	NAS	NAS UCI1 fue 53.66 (IC 95%; 51.81-55.52) y UCI2 fue 55.81 (IC 95%: 54.08 - 57.54)	12.8 días de estancia en UCI1 y 13.17 en UCI2 10.8% mortalidad UCI1 y 13.5% mortalidad UCI2
Carmona - Monge y col, 2013^19^.	563 pacientes	Descriptivo prospectivo	NAS	NAS total fue de 65.9 ±6.6; 61.6±8.1 para el SCA, 66.3 ±2.4 para IRA y 69.9 ±4.8 para sepsis	3.3 día s fue la mediana de la estancia 9.2% de mortalidad
Andrade y col, 2006^20^.	50 pacientes	Descriptivo prospectivo	NAS	El promedio del NAS fue de 66.5± 9.1	3.5 días de estancia
Grillo - Padilha y col, 2015^21^.	758 pacientes	Estudio transversal multicéntrico	NAS	El NAS fue del 72.8% en promedio, oscilando entre el 44.5% (España) y el 101.8% (Noruega). Mientras que para Polonia, Grecia y Egipto fue 83.0%, 64.6% y 57.1%, respectivamente	4.4±6.2 días de estanc ia 8.2% de mortalidad
Inoue y col, 2008^22^.	107 pacientes	Estudio descriptivo y exploratorio	NAS	La carga de trabajo diaria para el equipo de enfermería fue 697.3 ± 83.5	N A
Ducci y col, 2008^23^.	104 pacientes	Estudio de desarrollo metodológico.	NAS	Promedio de NAS aplicado de manera prospectivo fue 59.8 ±12.1 y de forma retrospectiva fue 52.7± 9.2	N A
Leite y col , 2012^24^.	66 pacientes	Descriptivo cuantitativo, retrospectivo	NAS	El promedio total de NAS fue del 68.1%, mínimo del 51.5% y máximo 108.3%	N A
Goulart y col, 2017^25^.	529 pacientes	Estudio longitudin al	NAS	El promedio del NAS fue 6 4.5%±8.2, siendo al ingreso de 78.0±6.6 y 68.9±8.8 al egreso.	7.3±9.2 días de estanc ia 12.5% mortalidad
Lucchini y col, 2013^26^.	5856 pacientes	Estudio observacional, retrospectivo	NAS	El promedio general para todos los pacientes fue del 65.97%±2.53, de 72,55%± 16.28 para la Unidad de Cuidados Intensivos Generales, del 59.33%± 16.54 para Unidad de Cuidados Intensivos Neuroquirúrgicos y de 63,51%±14.69 para la Unidad de Cuidados Intensivos Cardiotorácicos.	4.82± 8. 68 días de estancia
Souza y col, 2014^27^.	200 pacientes	Corte transversal	NAS	El promedio del NAS fue 71.3%±16.9	El sexo, la presencia de insuficien cia pulmonar, el número de regiones corporales lesionadas y el riesgo de muerte según el Nivel II de Fisiología Aguda Simplificada fueron asociados con un alto grado de carga de trabajo
Queijo y col, 2013^28^.	100 pacientes	Corte transversal	NAS	La NAS promedio fue del 65.18%±6.63%	5.10 ±= 4,90 días de estancia
Daud-Gallotti y col, 2012^29^.	195 pacientes	Cohorte prospectiva	NAS	El NAS fue 81.2± 16.2 en los pacientes que presentaron infecciones asociadas al cuidado de salud en comparar con el 66.7±20.3 en pacientes que no presentaron.	NA
Yatsue-Conishi y col, 2006^30^.	50 pacientes	Descriptivo prospectivo	NAS	El promedio para los pacientes de permanencia completa más incompleta fue de 65.5± 18.8, de 49.5± 10.5 incompleta y 69.6± 18.2 para completa.	17±20.4 días de estancia 18.7 mortalidad
TISS-28					
Autor/Año	Población (n)	Diseño	Método	Resultado	Otras variables de interés
Lapichino, 1991^31^.	28 pacientes	Estudio prospectivo	TOSS (Sistema de puntaje orientado al tiempo)	El promedio de TISS fue 29.9±0.2 puntos y TOSS 712± 3 min.	9.1±0.2 días de estancia media 23.0% de mortalidad
Grillo-Padilha y col, 2007^32^.	271 pacientes	Estudio Prospectivo	TISS-28	El promedio de TISS-28 fue 23 puntos (rango: 14-32 puntos).	7.7±10.4 días de la estadía 25.0% de mortalidad
Malstam y col, 1992^33^.	2693 pacientes	Corte trasversal	TISS modificado	El promedio de TISS fue de 114 ±218, para los pacientes críticamente enfermos (TISS Clase IV) y la carga fue de 437±401 puntos, mientras los pacientes que fallecieron mostraron un puntaje TISS promedio de 239 ± 364.	4.5±8.9 días de estancia 9.7% mortalidad
Mariusz- Wysokinskiy col, 2010^34^.	155 pacientes	Estudio Transversal	TISS-28	El promedio general de TISS-28 para las 3 UCIs fue de 38.64. Mientras que el promedio 39.63± 12.27 para la UCI de tipo 3, 37.17±9.54 para la UCI de tipo 2 y 39.32± 7.67 para UCI tipo 1.	25.03 días (r ango:1-190) de estancia
Kiekkas y col, 2008^35^.	396 pacientes	Observacional prospectivo	TISS-28	El promedio de 23.5 TISS-28 puntos. Los valores de mediana de carga de trabajo de enfermería variaron de 20.5-35.8 TISS-28 puntos por enfermera.	9.2 ± 15.9 días de estancias
NEMS					
Autor/Año	Población (n)	Diseño	Método	Resultado	Otras variables de interés
Barroso y col,	600 pacientes	Descriptivo	NEMS	El promedio del NEMS fue 22.75 ± 8.60 para el	NA
Autor/Año	Población (n)	Diseño	Método	Resultado	Otras variables de interés
Morini-Altafin y col, 2014^37^.	437 pacientes	Estudio Longitudinal y prospectivo.	NAS y TISS-28	El promedio general del NAS fue 74.47±8.77 y 87.54±8.26 al ingreso mientras que para el TISS-28 fue 25.78±6.64 y 25.77±7.19 al ingreso.	9.16±11.35 días de estancia 41.0% mortalidad
Grillo-padilha y col, 2008^38^.	200 pacientes	Estudio exploratorio descriptivo y prospectivo	NAS y TISS	El promedio del NAS fue 67.2% (min: 54.3- max: 107.2%). El promedio de TISS-28 fue 24.2±7.6	4.6±5dias de estancia 17.5% mortalidad
Rothen y col, 1999^39^.	2743 pacientes	Corte trasversal	NEMS y TISS-28	El promedio de NEMS fue 26.0 ± 8.1 y para el TISS-28 fue 26.5 ± 7.9.	76 ± 140 días de estancia 8.7% mortalidad
Adell y col, 2005^40^.	366 pacientes	Descriptivo longitudinal	NEMS y NAS	El promedio de NAS global fue 41.27 ± 10.80 mientras que NAS durante el día fue 50.40±15.29 y para el NEMS fue de 27.52±8.01	15.3% de mortalidad 5.2 ± 7.6 días de estancia
Stafseth y col, 2011^41^.	235 pacientes	Diseño descriptivo exploratorio	NEMS y NAS	La media NEMS fue de 32.7 ± 8.98 y la media NAS fue 96,24%±22,35%	5 días promedio de estancia
Carmona-Monge y col, 2013^42^.	730 pacientes	Prospectivo correlacional	NEMS y NAS	EL NAS promedio total fue 707.57 y 280.39 para el NEMS.	3.13 promedio de estancia 12.5% mortalidad
Kraljic y col, 2017^43^.	99 pacientes	Estudio Prospectivo	NEMS y NAS	El valor de NAS por paciente en el turno de día fue de 77.3±13.6, mientras que el valor acumulado en turnos diurnos fue 251.1 ±110.5, para el NEMS en turnos diarios fue 93.2±40.2	NA
Braña y col, 2007^44^.	91 pacientes	Descriptivo y retrospectivo	Escala de Valoración de las Cargas de trabajo y Tiempos de Enfermería (VACTE©) y NEMS	El promedio del NEMS fue de 19.5±5.7, mientras que para el VACTE© la media fue de 365± 91.2	NA
Duccy y col, 2008^45^.	55 pacientes	Descriptivo correlacional	NAS, TISS-28 y NEMS.	La carga de trabajo de enfermería medida por NAS 73.7%, de 66.2% para el TISS-28 y 59.7% para el NEMS	7.4% mortalidad
Valls-Matarín y col,2014^46^	217 pacientes	Estudio descriptivo transversal.	NAS, NEMS y VACTE.	El puntaje promedio total fue: NAS: 696.8 , NEMS: 311.8 (55.3) y VACTE: 4.978 .	NA
OTROS MÉTODOS					
Autor/Año	Población (n)	Diseño	Método	Resultado	Otras variables de interés
Adomat y col, 2003^47^.	21 pacientes	Estudio observacional	En este estudio, se utilizó un grabador de video para documen tar la actividad de la enfermera durante 48 cambios continuos en dos unidades de cuidados intensivos	Las enfermeras pasaron menos tiempo con los pacientes categorizados como en necesidad de cuidados intensivos que aquellos que necesitan alta dependencia cuidado en ambas unidades.	NA
Guo y col, 2016^48^.	6 pacientes	Descriptivo	Sistema de seguimien to Automatizado de la Actividad Clínica (CATS), para controlar y evaluar la actividad clínica en la cabecera del paciente	Se encontró que la intervención promedio en 24 horas es de 22.0 minutos/hora. La interven ción promedio durante el día (de 7 a. M. A 11 p. M.) fue de 23.6 minutos/hora, 1.4 veces más alta que las 11 p.m. -7 a.m. que es de 16.8 minutos/hora.	NA

En una reciente actualización de la literatura, se encontró que el NAS, por lo menos para Lati noamérica, continúa siendo el instrumento más utilizado para la evaluación de la carga laboral en enfermería^49^. Incluso, se han realizado varias adaptaciones del NAS en países como Chile y Colombia entre otros^50^^), (^^51^. De hecho, el presente grupo investigador ha tenido la experiencia de publicar dos estudios de tipo descriptivo correlacional en donde se aplicó este instrumento^52^^), (^^53^.

Para finalizar, es importante recordar la importancia que tiene la medición de la carga labo ral, independientemente del instrumento que para ello se use, pues su evaluación contribuye, como ya se ha descrito, en la seguridad del paciente y la calidad de la atención y a su vez, tienen un impacto en la propia salud de los profesionales de enfermería, quienes a raíz de la pandemia por COVID-19 se han enfrentado a cambios laborales, personales y sociales, con lo que cada vez se hace más evidente la necesidad de evaluar la carga laboral con instrumentos que se susten ten en evidencia científica^54^.

## Conclusiones

Se identificaron diferentes herramientas para medir la carga de trabajo en Enfermería en las UCIs. La más utilizada es el NAS, el cual cuenta con estudios de validación y aplicación suficiente para poder ser aplicadas en diferentes entornos culturales. De igual manera el TISS-28 y el NEMS son otras de las herramientas más usadas. Se debe continuar los procesos de validación y eva luación de estas herramientas en diferentes tipos de UCI.

Además, durante la revisión de este estudio no se presentaron limitaciones al momento de la búsqueda de fuentes bibliográficas, sin embargo, al estructurar el siguiente estudio, se logra percibir que son pocas las herramientas que existen para evaluar la carga de trabajo de Enfer meras en UCIs, razón por la cual se hace necesario generar interés en los investigadores para la actualización o creación de estas.
